# Endocrine and Growth Abnormalities in 4H Leukodystrophy Caused by Variants in *POLR3A*, *POLR3B*, and *POLR1C*

**DOI:** 10.1210/clinem/dgaa700

**Published:** 2020-10-01

**Authors:** Félixe Pelletier, Stefanie Perrier, Ferdy K Cayami, Amytice Mirchi, Stephan Saikali, Luan T Tran, Nicole Ulrick, Kether Guerrero, Emmanouil Rampakakis, Rosalina M L van Spaendonk, Sakkubai Naidu, Daniela Pohl, William T Gibson, Michelle Demos, Cyril Goizet, Ingrid Tejera-Martin, Ana Potic, Brent L Fogel, Bernard Brais, Michel Sylvain, Guillaume Sébire, Charles Marques Lourenço, Joshua L Bonkowsky, Coriene Catsman-Berrevoets, Pedro S Pinto, Sandya Tirupathi, Petter Strømme, Ton de Grauw, Dorota Gieruszczak-Bialek, Ingeborg Krägeloh-Mann, Hanna Mierzewska, Heike Philippi, Julia Rankin, Tahir Atik, Brenda Banwell, William S Benko, Astrid Blaschek, Annette Bley, Eugen Boltshauser, Drago Bratkovic, Klara Brozova, Icíar Cimas, Christopher Clough, Bernard Corenblum, Argirios Dinopoulos, Gail Dolan, Flavio Faletra, Raymond Fernandez, Janice Fletcher, Maria Eugenia Garcia Garcia, Paolo Gasparini, Janina Gburek-Augustat, Dolores Gonzalez Moron, Aline Hamati, Inga Harting, Christoph Hertzberg, Alan Hill, Grace M Hobson, A Micheil Innes, Marcelo Kauffman, Susan M Kirwin, Gerhard Kluger, Petra Kolditz, Urania Kotzaeridou, Roberta La Piana, Eriskay Liston, William McClintock, Meriel McEntagart, Fiona McKenzie, Serge Melançon, Anjum Misbahuddin, Mohnish Suri, Fernando I Monton, Sebastien Moutton, Raymond P J Murphy, Miriam Nickel, Hüseyin Onay, Simona Orcesi, Ferda Özkınay, Steffi Patzer, Helio Pedro, Sandra Pekic, Mercedes Pineda Marfa, Amy Pizzino, Barbara Plecko, Bwee Tien Poll-The, Vera Popovic, Dietz Rating, Marie-France Rioux, Norberto Rodriguez Espinosa, Anne Ronan, John R Ostergaard, Elsa Rossignol, Rocio Sanchez-Carpintero, Anna Schossig, Nesrin Senbil, Laura K Sønderberg Roos, Cathy A Stevens, Matthis Synofzik, László Sztriha, Daniel Tibussek, Dagmar Timmann, Davide Tonduti, Bart P van de Warrenburg, Maria Vázquez-López, Sunita Venkateswaran, Pontus Wasling, Evangeline Wassmer, Richard I Webster, Gert Wiegand, Grace Yoon, Joost Rotteveel, Raphael Schiffmann, Marjo S van der Knaap, Adeline Vanderver, Gabriel Á Martos-Moreno, Constantin Polychronakos, Nicole I Wolf, Geneviève Bernard

**Affiliations:** 1 Department of Neurology and Neurosurgery, McGill University, Montreal, QC, Canada; 2 Department of Pediatrics, McGill University, Montreal, QC, Canada; 3 Department of Human Genetics, McGill University, Montreal, QC, Canada; 4 Child Health and Human Development Program, Research Institute of the McGill University Health Centre, Montreal, QC, Canada; 5 Department of Specialized Medicine, Division of Medical Genetics, McGill University Health Centre, Montreal, QC, Canada; 6 Division of Child Neurology, Department of Pediatrics, CHU Sainte-Justine, Université de Montréal, Montreal, QC, Canada; 7 Department of Child Neurology, Amsterdam Leukodystrophy Center, Emma Children’s Hospital, Amsterdam University Medical Centers, and Amsterdam Neuroscience, Vrije Universiteit Amsterdam, Amsterdam, The Netherlands; 8 Center of Biomedical Research, Faculty of Medicine, Diponegoro University, Semarang, Indonesia; 9 Department of Pathology, Centre Hospitalier Universitaire de Québec, Québec City, QC, Canada; 10 Division of Neurology, Children’s Hospital of Philadelphia, Philadelphia, PA, USA; 11 Department of Clinical Genetics, Amsterdam UMC, Vrije Universiteit Amsterdam, Amsterdam, The Netherlands; 12 Department of Neurogenetics, Kennedy Krieger Institute, Johns Hopkins Medical Institutions, Baltimore, MD, USA; 13 Division of Neurology, Children’s Hospital of Eastern Ontario, University of Ottawa, Ottawa, ON, Canada; 14 Department of Medical Genetics, University of British Columbia, BC Children’s Hospital Research Institute, Vancouver, BC, Canada; 15 Division of Neurology, Department of Pediatrics, University of British Columbia, BC Children’s Hospital, Vancouver, BC, Canada; 16 Centre de Référence Neurogénétique, Service de Génétique Médicale, Bordeaux University Hospital, and Laboratoire MRGM, INSERM U1211, Université de Bordeaux, Bordeaux, France; 17 Department of Neurology, Hospital Universitario Nuestra Señora de Candelaria, 38010 Santa Cruz de Tenerife, Canary Islands, Spain; 18 Department of Neurology, Clinic for Child Neurology and Psychiatry, Medical Faculty University of Belgrade, Belgrade, Serbia; 19 Departments of Neurology and Human Genetics, David Geffen School of Medicine, University of California, Los Angeles, CA, USA; 20 Montreal Neurological Institute, Montreal, QC, Canada; 21 Centre Mère Enfant, CHU de Québec, Québec City, QC, Canada; 22 Department of Pediatrics, Université de Sherbrooke, Sherbrooke, QC, Canada; 23 Faculdade de Medicina, Centro Universitario Estácio de Ribeirão Preto, Ribeirão Preto, SP, Brazil; 24 Department of Pediatrics, University of Utah School of Medicine, Salt Lake City, UT, USA; 25 Department of Paediatric Neurology, Erasmus University Hospital - Sophia Children’s Hospital, 3015 CN Rotterdam, The Netherlands; 26 Neuroradiology Department, Centro Hospitalar do Porto, Porto, Portugal; 27 Department of Paediatric Neurology, Royal Belfast Hospital for Sick Children, Belfast, UK; 28 Division of Pediatrics and Adolescent Medicine, Oslo University Hospital, Ullevål, 0450 Oslo, and University of Oslo, Oslo, Norway; 29 Department of Pediatrics, Emory School of Medicine, Atlanta, GA, USA; 30 Department of Medical Genetics, Children’s Memorial Health Institute, Warsaw, Poland; 31 Department of Pediatrics, Medical University of Warsaw, Warsaw, Poland; 32 Department of Child Neurology, University Children’s Hospital Tübingen, Tübingen, Germany; 33 Department of Child and Adolescent Neurology, Institute of Mother and Child, Warsaw, Poland; 34 Center of Developmental Neurology (SPZ Frankfurt Mitte), Frankfurt, Germany; 35 Peninsula Clinical Genetics Service, Royal Devon and Exeter NHS Foundation Trust, Exeter, UK; 36 Division of Genetics, Department of Pediatrics, School of Medicine, Ege University, Izmir, Turkey; 37 Division of Neurology, Department of Pediatrics, Children’s Hospital of Philadelphia, Philadelphia, PA, USA; 38 Division of Pediatric Neurology, Department of Neurology, UC Davis Health System, Sacramento, CA, USA; 39 Department of Pediatric Neurology and Developmental Medicine, Dr. v. Hauner Children’s Hospital, University Hospital, LMU Munich, Munich, Germany; 40 University Children’s Hospital, University Medical Center Hamburg-Eppendorf, Hamburg, Germany; 41 Department of Child Neurology, University Children’s Hospital Zurich, Zurich, Switzerland; 42 Metabolic Clinic, Women’s and Children’s Hospital, North Adelaide, South Australia, Australia; 43 Department of Child Neurology, Thomayers Hospital, Prague, Czech Republic; 44 Department of Neurology, Povisa Hospital, Vigo, Spain; 45 Department of Neurology, King’s College Hospital, London, UK; 46 Division of Endocrinology & Metabolism, Department of Medicine, University of Calgary, Calgary, AB, Canada; 47 Third Department of Pediatrics, National and Kapodistrian University of Athens, “Attikon” Hospital, Athens, Greece; 48 Bristow Pediatrics, Bristow, VA, USA; 49 Institute for Maternal and Child Health, IRCCS Burlo Garofolo, Trieste, Italy; 50 Pediatric Neurology Associates, Tampa, FL, USA; 51 Genetics and Molecular Pathology, Women’s and Children’s Hospital, Adelaide, South Australia, Australia; 52 Department of Neurology, The Royal London Hospital, London, UK; 53 Institute for Maternal and Child Health, IRCCS Burlo Garofolo, 34100 Trieste, and University of Trieste, Trieste, Italy; 54 Division of Neuropaediatrics, Hospital for Children and Adolescents, University Leipzig, Leipzig, Germany; 55 Neurogenetics Unit, Department of Neurology, Hospital JM Ramos Mejia, ADC, Buenos Aires, Argentina; 56 Department of Child Neurology, Indiana University, Indianapolis, IN, USA; 57 Department of Neuroradiology, University Hospital Heidelberg, Heidelberg, Germany; 58 Department of Child Neurology, Vivantes Klinikum, Berlin, Germany; 59 Department of Pediatrics, University of British Columbia, Vancouver, BC, Canada; 60 Nemours Biomedical Research, Nemours/Alfred I. duPont Hospital for Children, Wilmington, DE, USA; 61 Department of Medical Genetics and Alberta Children’s Hospital Research Institute, University of Calgary, Calgary, AB, Canada; 62 Neurogenetics Unit, Department of Neurology, Hospital JM Ramos Mejia and CONICET, ADC, Buenos Aires, Argentina; 63 Molecular Diagnostics Laboratory, Nemours/Alfred I. duPont Hospital for Children, Wilmington, DE, USA; 64 PMU Salzburg, 5020 Salzburg, Austria; Clinic for Neuropediatrics and Neurorehabilitation, Epilepsy Center for Children and Adolescents, Schön Klinik Vogtareuth, Vogtareuth, Germany; 65 Department of Child Neurology, Kantonsspital Luzern, Luzern, Switzerland; 66 Department of Child Neurology, University Children’s Hospital Heidelberg, Heidelberg, Germany; 67 Department of Neuroradiology, Montreal Neurological Institute and Hospital, McGill University, Montreal, QC, Canada; 68 Division of Clinical and Metabolic Genetics, The Hospital for Sick Children, Toronto, ON, Canada; 69 Pediatric Specialists of Virginia, Fairfax, VA, USA; 69a Department of Neurology, Children’s National Medical Center, Washington, DC, USA; 70 South West Thames Regional Genetics Service, St. George’s Hospital, London, UK; 71 Genetic Services of Western Australia, Subiaco, WA, Australia; 71a School of Paediatrics and Child Health, University of Western Australia, Perth, WA, Australia; 72 Department of Medical Genetics, McGill University Health Centre, Montreal Children’s Hospital, Montreal, QC, Canada; 73 Essex Centre for Neurological Sciences, Queen’s Hospital, Romford, UK; 74 Nottingham Clinical Genetics Service, City Hospital Campus, Nottingham University Hospitals NHS Trust, Nottingham, UK; 75 Service de Génétique Médicale, CHU de Bordeaux, Bordeaux, France; 76 Department of Neurology, Tallaght University Hospital, Tallaght, Ireland; 77 Department of Pediatrics, University Medical Center Hamburg-Eppendorf, Hamburg, Germany; 78 Department of Medical Genetics, Ege University, Izmir, Turkey; 79 Child Neurology and Psychiatry Unit, IRCCS Mondino Foundation, Pavia, Italy; 80 Department of Pediatrics, Subdivision of Pediatric Genetics, Faculty of Medicine, Ege University, Izmir, Turkey; 81 Children’s Hospital St. Elisabeth and St. Barbara, Halle (Saale), Germany; 82 Department of Pediatrics, The Joseph M. Sanzari Children’s Hospital, Hackensack University Medical Center, Hackensack, NJ, USA; 83 Clinic for Endocrinology, Diabetes and Diseases of Metabolism, University Clinical Center, Belgrade & School of Medicine, University of Belgrade, Belgrade, Serbia; 84 Hospital Sant Joan de Deu, Passeig de Sant Joan de Deu nº2, Barcelona, Spain; 85 Department of Neurology, Children’s Hospital of Philadelphia, Philadelphia, PA, USA; 85a Department of Genetics, MetroHealth Hospital, Cleveland, OH, USA; 86 Department of Pediatrics and Adolescent Medicine, Division of General Pediatrics, Medical University of Graz, Graz, Austria; 87 Department of Pediatric Neurology, Emma Children’s Hospital, 1105 Amsterdam, The Netherlands; 88 Medical Faculty, University of Belgrade, Belgrade, Serbia; 89 Department of Paediatric Neurology, University Children’s Hospital, Heidelberg, Germany; 90 Centre Hospitalier Universitaire de Sherbrooke - Hôpital Fleurimont, Sherbrooke, QC, Canada; 91 Hunter New England LHD, University of Newcastle, NSW, Australia; 92 Centre for Rare Diseases, Aarhus University Hospital, Aarhus, Denmark; 93 Departments of Neurosciences and Pediatrics, CHU-Sainte-Justine, Université de Montréal, Montreal, QC, Canada; 94 Pediatric Neurology Unit, Department of Pediatrics, Clinica Universidad de Navarra, Pamplona, Spain; 95 Institute of Human Genetics, Medical University Innsbruck, Innsbruck, Austria; 96 Department of Child Neurology, Kırıkkale University Medical Faculty, Kırıkkale, Turkey; 97 Applied Human Molecular Genetics, Kennedy Center, Copenhagen University Hospital, Rigshospitalet, Glostrup, Denmark; 98 Department of Pediatrics, Division of Medical Genetics, University of Tennessee College of Medicine, Chattanooga, TN, USA; 99 Department of Neurodegeneration, Hertie Institute for Clinical Brain Research and Centre of Neurology, German Research Center for Neurodegenerative Diseases (DZNE), University of Tübingen, Tübingen, Germany; 100 Department of Paediatrics, University of Szeged, Szeged, Hungary; 101 Department of General Pediatrics, Neonatology and Pediatric Cardiology, University Children’s Hospital, Heinrich-Heine-University, 40225 Düsseldorf, Germany; 102 Department of Neurology, Essen University Hospital, University of Duisburg-Essen, Essen, Germany; 103 Child Neurology Unit, V. Buzzi Children’s Hospital, Milano, Italy; 104 Department of Neurology, Donders Institute for Brain, Cognition, and Behaviour, Radboud University Medical Centre, Nijmegen, The Netherlands; 105 Sección Neuropediatría. Hospital Maternoinfantil Gregorio Marañón, Madrid, Spain; 106 Division of Neurology, Department of Pediatrics, Children’s Hospital of Eastern Ontario, Ottawa, ON, Canada; 107 Department of Neuroscience and Rehabilitation, The Sahlgrenska Academy, University of Gothenburg, Gothenburg, Sweden; 108 Paediatric Neurology, Birmingham Children’s Hospital, Birmingham, UK; 109 T. Y. Nelson Department of Neurology and Neurosurgery and the Institute for Neuroscience and Muscle Research, The Children’s Hospital at Westmead, Sydney, New South Wales, Australia; 110 Department of Pediatric Neurology, University Hospital Kiel, Germany; 110a Neuropediatrics Section of the Department of Pediatrics, Asklepios Clinic Hamburg Nord-Heidberg, Hamburg, Germany; 111 Division of Clinical and Metabolic Genetics, Division of Neurology, Department of Pediatrics, The Hospital for Sick Children, University of Toronto, Toronto, ON, Canada; 112 Emma Children’s Hospital, Amsterdam UMC, Pediatric Endocrinology, Vrije Universiteit Amsterdam, Amsterdam, The Netherlands; 113 Institute of Metabolic Disease, Baylor Scott & White Research Institute, Dallas, TX, USA; 114 Department of Functional Genomics, Center for Neurogenomics and Cognitive Research, VU University, Amsterdam, The Netherlands; 115 Department of Neurology, Perelman School of Medicine, University of Pennsylvania, Philadelphia, PA, USA; 116 Department of Pediatric Endocrinology, Hospital Infantil Universitario Niño Jesús, Instituto de Investigación La Princesa, Madrid, Spain; 117 Department of Pediatrics, Universidad Autónoma de Madrid, 28049 Madrid, Spain; 117a CIBER de Fisiopatologia de la Obesidad y Nutriciόn (CIBEROBN), Instituto de Salud Carlos III, Madrid, Spain; 118 Division of Endocrinology, Montreal Children’s Hospital and the Endocrine Genetics Lab, Research Institute of the McGill University Health Centre, Montreal, QC, Canada

**Keywords:** POLR3-related leukodystrophy, 4H leukodystrophy, hypomyelination, hypogonadotropic hypogonadism

## Abstract

**Context:**

4H or POLR3-related leukodystrophy is an autosomal recessive disorder typically characterized by hypomyelination, hypodontia, and hypogonadotropic hypogonadism, caused by biallelic pathogenic variants in *POLR3A*, *POLR3B, POLR1C*, and *POLR3K*. The endocrine and growth abnormalities associated with this disorder have not been thoroughly investigated to date.

**Objective:**

To systematically characterize endocrine abnormalities of patients with 4H leukodystrophy.

**Design:**

An international cross-sectional study was performed on 150 patients with genetically confirmed 4H leukodystrophy between 2015 and 2016. Endocrine and growth abnormalities were evaluated, and neurological and other non-neurological features were reviewed. Potential genotype/phenotype associations were also investigated.

**Setting:**

This was a multicenter retrospective study using information collected from 3 predominant centers.

**Patients:**

A total of 150 patients with 4H leukodystrophy and pathogenic variants in *POLR3A, POLR3B,* or *POLR1C* were included.

**Main Outcome Measures:**

Variables used to evaluate endocrine and growth abnormalities included pubertal history, hormone levels (estradiol, testosterone, stimulated LH and FSH, stimulated GH, IGF-I, prolactin, ACTH, cortisol, TSH, and T4), and height and head circumference charts.

**Results:**

The most common endocrine abnormalities were delayed puberty (57/74; 77% overall, 64% in males, 89% in females) and short stature (57/93; 61%), when evaluated according to physician assessment. Abnormal thyroid function was reported in 22% (13/59) of patients.

**Conclusions:**

Our results confirm pubertal abnormalities and short stature are the most common endocrine features seen in 4H leukodystrophy. However, we noted that endocrine abnormalities are typically underinvestigated in this patient population. A prospective study is required to formulate evidence-based recommendations for management of the endocrine manifestations of this disorder.

Leukodystrophies are a group of rare genetic diseases characterized by abnormal white matter in the central nervous system (CNS), which often result in progressive neurodegeneration and premature death (1, 2). Based on whether the white matter abnormalities seen on brain magnetic resonance imaging (MRI) are caused by insufficient initial myelin deposition or altered myelin homeostasis, leukodystrophies can be classified as hypomyelinating or non-hypomyelinating, respectively (3-5). 4H leukodystrophy, also known as RNA polymerase III (POLR3)-related leukodystrophy, is an autosomal recessive hypomyelinating leukodystrophy associated with several characteristic neurological and non-neurological clinical features, primarily including hypomyelination, hypodontia, and hypogonadotropic hypogonadism (6). Commonly presenting neurological signs include cerebellar manifestations, such as ataxia and dysmetria, as well as pyramidal, extrapyramidal, and cognitive features (5, 7). Non-neurological manifestations can include myopia and endocrine features such as growth hormone (GH) deficiency and short stature (7-12). 4H leukodystrophy is also typically associated with a unique MRI phenotype, including cerebellar atrophy, progressive thinning of the corpus callosum, and diffuse hypomyelination with relative preservation of specific structures, namely the dentate nucleus, optic radiations, anterolateral nucleus of the thalamus, globus pallidus, and corticospinal tracts at the level of the posterior limb of the internal capsule (13, 14). 4H leukodystrophy has been found to be caused by biallelic pathogenic variants in *POLR3A, POLR3B, POLR1C*, and *POLR3K,* each of which encode subunits of the POLR3 complex (15-18).

As this class of leukodystrophies has been discovered relatively recently, secondary features that are typically associated with this phenotype have not been comprehensively described. This study presents a thorough investigation of the endocrine and growth abnormalities associated with 4H leukodystrophy through an international cross-sectional retrospective study of 150 patients with a molecular confirmation of the diagnosis. Moreover, this study provides insight on the endocrine disorders associated with this disease, and how some endocrine abnormalities may have an impact on patients’ quality of life, thus highlighting the importance of considering endocrine therapeutic options and the associated impact on medical care.

## Materials and Methods

### Patients and study design

An international cross-sectional study was performed between 2015 and 2016 on a cohort of 150 patients (76 females, 74 males) with biallelic pathogenic variants in *POLR3A, POLR3B,* or *POLR1C* and hypomyelination on brain MRI. Chart review focused on endocrine data, including any available hormonal investigations and pubertal history information, as well as growth data, including height and head circumference. Other clinical features, including age at disease onset, genotype, and both neurological and non-neurological features were reviewed. This project was approved by the research ethics committee of the Montreal Children’s Hospital (11-105-PED), Canada; the Children’s Hospital of Philadelphia, USA; and the VU University Medical Center in Amsterdam, Netherlands. Informed written consent was obtained from all participants or their legal guardians. Several patients have been previously published in studies describing the genetic basis of the disease or in the delineation of other clinical features.

### Pubertal status

In females, to evaluate abnormalities in pubertal development, we primarily assessed age at menarche and considered puberty delayed if menarche had not occurred by the 16th birthday. We lacked information on breast development for most patients; however, for those that had information available, we evaluated Tanner stage of breast development at age 13 (persistence of Tanner stage 1). Of note, we considered menarche as the main criteria for evaluating abnormal puberty (i.e., if patients had delayed or absent menarche, this feature took priority over breast development stage). In males, we assessed Tanner staging for testicular growth, and considered puberty delayed if Tanner stage 1 for testicular growth persisted at age 14.

### Growth data

Data sets of patients’ height and head circumference at all available times from birth to their latest available visit were reviewed and standardized according to the appropriate sex and age references.

### Endocrine investigations

For patients whose information was available, we retrospectively reviewed levels of estradiol, testosterone, stimulated LH and FSH, stimulated GH, IGF-I, prolactin, ACTH, cortisol, TSH, and free T4.

### Statistical analysis

Descriptive statistics were produced for all studied parameters, including the median and minimum/maximum values for continuous variables and the count and percentage for categorical variables. For the latter, 95% confidence intervals (95% CI) were also produced using the normal approximation method.

For the growth analysis, the percentiles for each measure were determined using the World Health Organization growth charts. To compare height data accounting for age and sex, Z-scores were calculated using values and standard deviations from the World Health Organization child growth standards. Because 95% of the normal population are within 2 SDs from the mean, short stature was defined as a height value below 2 SDs for that corresponding age and sex. Similarly, for head circumference data, microcephaly was defined as a head circumference that was 2 SDs or more below the mean for that corresponding age and sex, whereas macrocephaly was defined as greater than 2 SDs above the mean.

The association between genotype and phenotype was assessed for exploratory purposes with the χ ^2^ test. Analyses were conducted using SPSS software, version 24 (Armonk, NY: IBM Corp.).

### Pituitary gland pathology

Pituitary gland pathology of one patient with 4H leukodystrophy and pathogenic variants in *POLR3A*, who had died of respiratory complications at age 19, was investigated via immunohistochemical staining of the adenohypophysis. Five-micrometer-thick vertico-frontal sections of the hypophysis were analyzed with immunostaining for FSH (Agilent catalog no. M3504, RRID: AB_2079146, dilution 1/400), LH (Agilent catalog no. M3502, RRID: AB_2135325, dilution 1/400), prolactin (Cell Marque catalog no. 210A-18, RRID: AB_1516984, dilution 1/250), ACTH (Agilent catalog no. M3501, RRID: AB_2166039, dilution 1/1000), TSH (Agilent catalog no. M3503, RRID:AB_2287785, dilution 1/300), and human growth hormone (HGH; Aglient catalog no. A0570, RRID:AB_2617170, dilution 1/3000).

## Results

### Molecular genetics and clinical features

Within our cohort of 150 patients, 56 had pathogenic variants in *POLR3A*, 81 in *POLR3B*, and 13 in *POLR1C*. Seventy-six patients were females and 74 were males (Table 1). Both neurological and non-neurological features were noted in the patient cohort. Many patients demonstrated ataxia (94%; 49/52), dysarthria (85%; 34/40), and dystonia (84%; 26/31). Other features can be seen in Table 1. Non-neurological features were also evident, including myopia (87%; 90/103) and teeth abnormalities (85%; 99/117).

**Table 1.** Patient Demographic Characteristics, Molecular Diagnosis, and Clinical and Endocrine Features

Characteristic	n (N)	Percentage
**Gender**		
Male	74 (150)	49.3%
Female	76 (150)	50.7%
**Molecular diagnosis**		
*POLR3A*	56 (150)	37.3%
*POLR3B*	81 (150)	54.0%
*POLR1C*	13 (150)	8.7%
**Clinical features** ^***a***^		
***Neurological***		
Ataxia	49 (52)	94.2%
Tremor	48 (66)	72.7%
Dystonia	26 (31)	83.9%
Dysarthria	34 (40)	85.0%
Dysphagia	18 (37)	48.7%
Sialorrhea	12 (25)	48.0%
Seizures	17 (61)	27.9%
***Non-neurological***		
Myopia	90 (103)	87.4%
Teeth abnormalities	99 (117)	84.6%
**Endocrine features**		
Short stature		
*Clinical impression*	57 (93)	61.3%
*Growth data*	53 (115)	46.1%
Delayed puberty		
*Clinical impression*	57 (74)	77.0%
*Tanner stage*	27 (32)	84.3%
Abnormal thyroid function	13 (59)	22.0%

n: number of identified patients per data sample. N: total number of patients in the data sample.

^*a*^N values vary as clinical data were not available for all 150 patients in the cohort.

### Reproductive hormones and pubertal development

#### Female patients.

Delayed puberty was reported in 89% (34/38; 95% CI, 80-99%) of female patients based on the clinical judgment of the treating physician. In analyzing our cohort based on the clinical information that was available for menarche and breast development, 21/25 patients (84%; 95% CI, 70-98%) were considered to have absent, delayed, or arrested puberty. Therefore, most females in our cohort presented with abnormal puberty and differing severities of early-onset hypogonadism.

**Figure 1.**
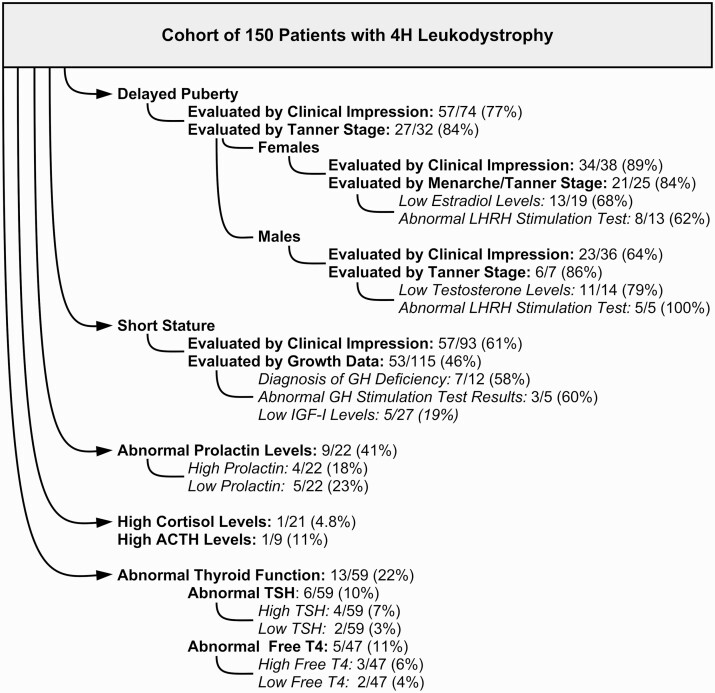
Summary of endocrine abnormalities in this cohort of patients with 4H leukodystrophy, according to the available clinical information on growth and endocrine features.

Of the female patients tested for specific hormone levels, 13/19 (68%; 95% CI, 48-89%) had low levels of estradiol. Luteinizing hormone releasing hormone (LHRH) stimulation tests were performed on 13 female patients, of whom 8 (62%; 95% CI, 35-88%) had abnormally low levels of both LH and FSH. In regard to sex steroid treatment, information was available for 29 patients, of whom 20 (69%) received treatment and 9 (31%) did not. Sex steroid medications induced menstruation and/or puberty in 9/20 (45%; 95% CI, 23-67%) female patients. In one patient, treatment was clearly ineffective, and in several cases, sex steroids were not well tolerated and caused adverse reactions. Of the untreated patients, 6/9 (67%) had abnormally low sex steroid levels, and of the 2/9 (22%) patients with normal levels, one had a clinical diagnosis of delayed puberty. Only 4 of 13 patients who had menarche experienced it spontaneously with no need for sex hormone treatment, at a median age of 12 years (minimum age, 11 years; maximum age, 13 years; n = 4). In patients who were treated with sex hormones, menarche occurred at a median age of 18 years (minimum age, 16 years; maximum age, 32 years; n = 9). For those who experienced treatment-induced menarche, information on spontaneous breast development was only available for 2 patients, who reached onset of breast development at ages 12 and 19 years old. Of the patients for which menarche did not yet occur, 3/4 (75%) were treated with sex hormones, one of which was only 16 years old at the time of data collection. The remaining patient who did not experience menarche had not been treated with sex hormones (1/4; 25%). Summarized results are shown in Fig. 1.

#### Male patients.

Overall, less information was available regarding puberty of males compared to females. Based on the physician’s clinical judgment, 23/36 (64%; 95% CI, 48-80%) male patients had delayed puberty. In analyzing our cohort based on the definition of delayed puberty (persistence of Tanner stage 1 for testicular growth at age 14), 6/7 (86%; 95% CI, 60-100%) were considered to have delayed puberty. Many patients lacked information on Tanner staging in adolescence and therefore could not be assessed for early pubertal abnormalities.

Of the male patients in our cohort tested for sex steroid levels, 11/14 (79%; 95% CI, 57-100%) had abnormally low testosterone levels. All patients with low testosterone also presented with delayed puberty (7/7, 100%). LHRH stimulation tests revealed abnormally low levels of LH in all patients that were tested (5/5, 100%). Information regarding sex steroid treatment was available for 23 male patients; approximately one-half of the patients (11/23; 48%) were treated, whereas 12/23 (52%) were not. Nearly all the males who received sex steroid medication were treated with testosterone (10/11, 91%), with the exception of one who was treated with chorionic gonadotropin (1/11, 9%). Treatment, however, was only effective in 5 patients (5/11; 45%; 95% CI, 16-75%), including 4 patients who received testosterone and the single patient who received chorionic gonadotropin treatment. Results are summarized in Fig. 1.

### Growth analysis

**Figure 2.**
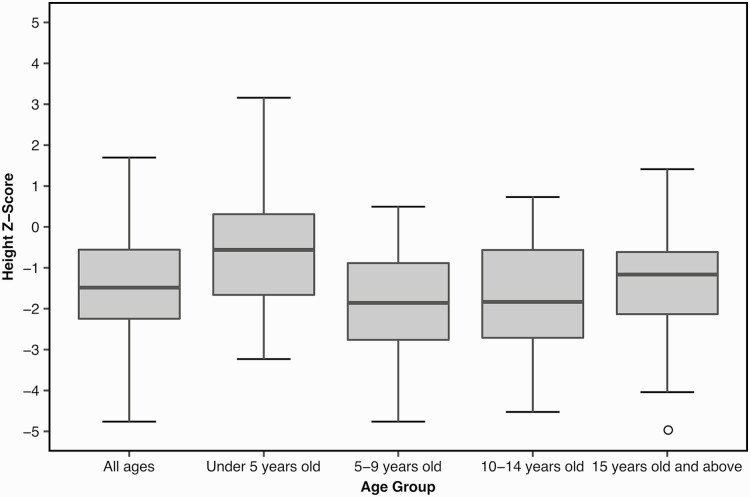
Box plots for Z-scores obtained for the mean height in different age categories. The height Z-score for the general population (mean value) is considered to be 0, and the clinical definition for short stature is two standard deviations below the mean (–2).

Linear growth data were obtained for 115 patients and analyzed using Z-scores. The Z-score for all ages ranged from –4.76 to 1.70 (median, –1.48). To determine whether growth was more severely affected in participants of certain ages, median Z-scores for heights of different age groups of patients were evaluated. For each age group range, an average Z-score was calculated for each patient based on all height data if multiple records were available. Some participants are represented in multiple age groups if records were available spanning different ranges. Z-scores for ages < 5 years (n = 42), 5 to 9 years (n = 37), 10 to 14 years (n = 28), and ≥ 15 years (n = 57) were found to be –0.56, –1.86, –1.83, and –1.16, respectively. Figure 2 shows boxplots of height Z-scores for all patients and for each age group. Across age groups, patients < 5 years of age had a median Z-score closest to that of the general population (0). Additionally, the < 5 years age group had a positive Z-score for the third quartile (0.31), whereas the other age groups (5-9 years, 10-14 years, and ≥ 15 years) each had negative third quartile values (–0.89, –0.57, and –0.62, respectively). Moreover, the maximum height Z-score of the < 5 years group (3.16) corresponds to the maximum value of the entire cohort, and the minimum Z-score for the < 5 years group (–3.23) is closest to 0 when comparing minimum values between all groups. These results suggest that the < 5 year age group seems to be least affected in regard to stature. In contrast, the 5 to 9 year age group is most affected as it has the lowest values for the maximum and median Z-score values of all groups. Moreover, within the 5 to 9 year age group, the median height Z-score was –1.86 and the Z-score of the first quartile was –2.76, where more than one-quarter of these patients had short stature.

Based on the clinical judgment of the treating physician, 57/93 patients (61%; 95% CI, 51-71%) were considered to have short stature. When analyzing the Z-scores of patients’ heights, 53/115 patients (46%; 95% CI, 37-55%) had values lower than 2 SDs and thus by clinical definition had short stature. Of 115 patients, 67 (58%; 95% CI, 49-67%) were also reported to have a height > –1.5 SD below the mean. Additionally, 68% of these patients (78/115; 95% CI, 59-76%) had a height lower than 1 SD below the mean. Thus, even if some patients did not meet the criteria for the clinical definition of short stature, our cohort seems to be smaller than the general population.

Head circumference data were analyzed using Z-scores in patients aged 0 to 5 years (n = 35). The Z-scores ranged from –2.93 to 2.44 (median, –0.04). Twenty patients (20/35, 57%; 95% CI, 41-74%) presented with a head circumference within 1 SD from the mean, whereas 31 patients (31/35, 89%; 95% CI, 78-99%) were within 2 SDs from the mean. Three patients (3/35, 9%; 95% CI, 0-18%) had values lower than 2 SDs below the mean and thus by clinical definition had microcephaly. Additionally, 14% of patients (5/35; 95% CI, 3-26%) were reported to have a head circumference lower than 1.5 SDs below the mean, and 34% (12/35; 95% CI, 19-50%) had a head circumference lower than 1 SD below the mean. One patient (1/35, 3%; 95% CI, 0-8%) met the criteria for macrocephaly, with a head circumference greater than 2 SDs above the mean.

Of our cohort of 150 patients, data regarding growth hormones were available for 12 patients, of whom 7 had a diagnosis of GH deficiency based on the clinical judgment of the treating physician (7/12, 58%; 95% CI, 30-86%). Of these 7 patients who were diagnosed with a GH deficiency, only 2 had a GH stimulation test, where one had a decreased response and the other presented with a normal response. Many patients only had a single measurement of GH; however, these results could not be analyzed as GH is secreted in a pulsatile manner, and therefore nonstimulated levels do not provide useful information for analysis. In total, 5 patients in our cohort had a GH stimulation test, in which 3 exhibited a decreased response (3/5, 60%; 95% CI, 17-100%), and the remaining 2 a normal response. Additionally, only 2 patients were treated with GH, which was ineffective in both cases. IGF-I values were only available for 27 patients, of which 19% presented with a low value (5/27, 19%; 95% CI, 4-33%). For those that had both a GH stimulation test and IGF-I levels measured, 2 patients had low levels of both tests, one had normal results for both, one had a normal GH stimulation test and low IGF-I levels, and one had a low GH stimulation test and normal IGF-I levels.

### Other endocrine abnormalities

In patients with abnormal prolactin levels (9/22, 41%; 95% CI, 20-61%), values were found to vary in both high and low ranges. Of these patients, 4 had high levels of prolactin (4/22, 18%; 95% CI, 2-34%), wherein 3 had levels at least 50% higher than normal (3/22, 14%; 95% CI, 0-28%). In contrast, 5 patients had low prolactin levels (5/22, 23%; 95% CI, 5-40%), of whom one had levels at least 50% lower than normal (1/22, 4.5%; 95% CI, 0-13%).

Nearly all patients who were tested for cortisol levels had results within the normal range (20/21, 95%; 95% CI, 86-100%). Additionally, most patients who were tested for ACTH levels displayed normal results (8/9, 89%; 95% CI, 68-100%). The single patient with abnormal cortisol levels presented with an elevated level (1/21, 5%; 95% CI, 0-14%), although his ACTH level was not tested. The single patient who displayed high ACTH levels (1/9, 11%; 95% CI, 0-32%) had normal cortisol levels.

Thyroid function was tested in 59 patients, where 13 showed abnormal results (13/59, 22%; 95% CI, 11-33%). Approximately 10% of the patients (6/59, 95% CI, 2-18%) had abnormal TSH levels, including 2 patients with low TSH levels (2/59, 3%; 95% CI, 0-8%), and 4 patients with high TSH levels (4/59, 7%; 95% CI, 0-13%). Of the 47 patients tested for free T4 levels, 5 showed abnormal results (5/47, 11%; 95% CI, 2-19%), including 3 with high levels (3/47, 6%; 95% CI, 0-13%), and 2 with low levels (2/47, 4%; 95% CI, 0-10%).

One patient with low TSH levels, but normal T4 levels, was diagnosed with subclinical hyperthyroidism. One patient had low T4 levels and high TSH levels, which is consistent with hypothyroidism. Additionally, one patient with an unknown TSH level, and a low free T4 level was diagnosed with hypothyroidism. Only one patient was treated with thyroid hormones, but no data were available regarding his hormonal levels. A summary of hormone levels is presented in Table 2.

**Table 2.** Summary of Hormonal Levels According to Mutated Gene

Hormone Levels		Genotype (No. of Patients)			All Patients
Hormone	Reported Levels	*POLR3A*	*POLR3B*	*POLR1C*	TOTAL
GH (stimulation test)	Low	2	0	1	3
	Normal	2	0	0	2
	% Abnormal	50.0%	0.0%	100.0%	60.0%
Prolactin	Low	3	2	0	5
	High	1	1	2	4
	Normal	8	3	2	13
	% Abnormal	33.3%	50.0%	50.0%	40.9%
TSH	Low	2	0	0	2
	High	1	2	1	4
	Normal	22	24	7	53
	% Abnormal	12.0%	7.7%	12.5%	10.2%
Free T4	Low	2	1	1	2
	High	0	2	1	3
	Normal	21	15	6	42
	% Abnormal	8.7%	16.7%	25.0%	10.6%

### Relationship between genotype and endocrine abnormalities

According to different genotypes (i.e., whether pathogenic variants were present in *POLR3A, POLR3B,* or *POLR1C*), the presence of delayed puberty and short stature in patients was analyzed as these features were most prevalent in our patient cohort. In terms of delayed puberty, significant differences were observed between genotypes (p < 0.001), with the highest incidence observed in patients with pathogenic variants in *POLR3A* (27/32, 84%; 95% CI, 72-97%), followed by those with variants in *POLR3B* (30/38, 79%; 95% CI, 66-92%). None of the patients with pathogenic variants in *POLR1C* (0/4; 0%) who had reached the appropriate age exhibited delayed puberty. Of the patients with pathogenic variants in *POLR3A,* 71% (22/31; 95% CI, 55-87%) had short stature, compared with 54% (32/59; 95% CI, 42-67%) with variants in *POLR3B*, and 100% (3/3) with variants in *POLR1C* (p = 0.113). Data on specific hormone measurements were limited, however, an analysis of levels of stimulated GH, prolactin, TSH, and free T4 between genotypes is also presented in Table 2.

### Pituitary gland pathology

**Figure 3.**
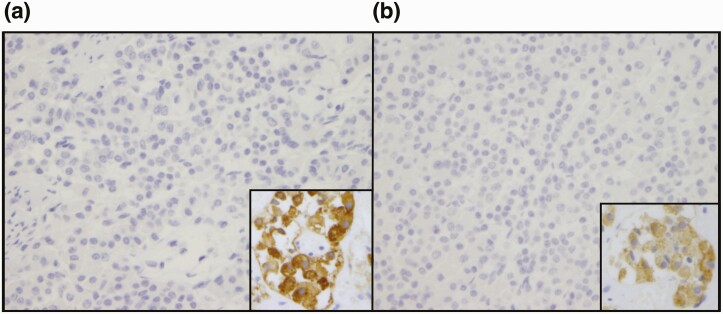
Immunohistochemical analysis of the anterior pituitary gland. Total lack of cytoplasmic expression of LH (A; left) and FSH (B; right) is seen relative to the external control (insert). Magnification ×400.

Pathological investigations were performed following autopsy of a 19-year-old patient who was homozygous for the *POLR3A* pathogenic variant c.2015G>A (p.G672E). Clinically, the patient did not show signs of puberty and was reported to have hypogonadotropic hypogonadism; however, results of specific hormone levels were not available. He also had short stature, falling in the 5th percentile for height at age 18 years. The patient demonstrated typical neurological features associated with 4H leukodystrophy, including ataxia with abnormal gait, tremor, dystonia, spasticity, and dysarthria. MRI scans revealed diffuse hypomyelination with cerebellar atrophy and a thin corpus callosum, consistent with the pattern for 4H leukodystrophy. He also had epilepsy, with complex partial seizures. Dentition was abnormal, with notable hypodontia. Ocular abnormalities included myopia, mild optic nerve atrophy, and esotropia. With age, chronic progressive decline in neurological function was evident, along with decline in motor ability. He was wheelchair bound at age 8 years, eventually becoming quadriplegic with increased tone in all extremities. He had dysphagia, with frequent choking episodes, and progressively lost the ability to eat unaided, further requiring a gastrostomy. The patient had recurrent aspiration pneumonias and died at the age of 19 from complications of bilateral pneumonia. Immunohistochemical analysis of the anterior pituitary gland revealed an absence of immunostaining of anti-FSH and anti-LH antibodies (Fig. 3) and normal immunostaining for anti-GH, anti-TSH, anti-prolactin, and anti-ACTH antibodies. This demonstrates an absence of secretion of gonadotropic hormones (FSH and LH) by the pituitary gland.

## Discussion

Our study confirms that endocrine impairment is frequent in patients with 4H leukodystrophy and although limited data were available for the entire cohort of patients, our results reveal notable information regarding abnormalities in the pituitary-gonadotrophic axis. Delayed puberty was a common finding in our patient population. However, LHRH stimulation tests were only performed on a small percentage of patients to confirm hypogonadotropic hypogonadism. It should be noted that baseline FSH and LH levels are not useful for the diagnosis of hypogonadotropic hypogonadism, and stimulation tests should be performed for an accurate result. There are currently no guidelines for the introduction of sex steroid treatment in patients with 4H leukodystrophy. Still, we would recommend LHRH stimulation tests to confirm the diagnosis before initiating treatment. Thus, a pediatric endocrinologist should be included in the multidisciplinary team assessing patients with 4H leukodystrophy.

Treatment of hypogonadotropic hypogonadism remains controversial in this patient population; although there are significant benefits, there are also associated risks. One significant treatment advantage is the promotion of bone health by influencing bone remodeling. Another potential benefit is the physical appearance in a period of life where being similar to his or her peers is important. Such a factor is typically not a consideration for severely neurologically impaired children. Treating hypogonadotropic hypogonadism would allow the development of secondary sexual characteristics associated with normal pubertal development, and also induce a growth spurt, which might enhance motor difficulties and behavioral problems. Currently, little information is available in the literature regarding this treatment and its effects on this patient group. It was previously suggested that the same principles of hormone replacement therapy used in patients with other forms of hypogonadotropic hypogonadism should be applied to patients with 4H leukodystrophy (10). To induce puberty, sex steroids (testosterone for boys and estrogen/progesterone for girls) are the first line of therapy for patients with hypogonadotropic hypogonadism. If fertility induction is intended, pulsatile GnRH therapy could be tried, however, may not be effective, as some individuals with hypogonadotropic hypogonadism respond poorly to short-term stimulation (19). In this case, treatment with recombinant gonadotrophins could provide an alternative option. In this cohort, sex steroid treatment did not appear to be effective in all cases, however, it could not be established by what criteria response was judged, how long treatment was pursued, and/or by what dose. Collecting additional prospective data on sex hormone treatment for delayed puberty would allow better ascertainment of these situations and allow clinicians to provide further informed recommendations. In the meantime, we recommend that the decision to initiate sex steroid treatment is approached on an individual basis, while weighing the benefits (e.g., bone health) and disadvantages (e.g., rapid growth spurt with motor regression), together with the overall health of the patient (e.g., well vs. severely impaired) and only after measure of abnormal sex hormone levels confirms the diagnosis.

Pathological investigations of an affected patient confirmed dysfunction in the sex steroid axis as an absence of FSH and LH secretion was observed in the anterior pituitary gland. Low response to LHRH stimulation tests, observed in 72% of our patients, also supports the hypophyseal origin (20). Thus, it is likely that abnormal levels of FSH and LH are a result of pituitary gland malfunction, resulting in central hypogonadism.

Limited data were available regarding stimulated GH levels in our cohort of patients. Based on our results, decreased GH secretion could be frequent in patients with 4H leukodystrophy; however, very few regularly had levels of stimulated GH measured. Moreover, a GH stimulation test is necessary for the diagnosis of GH deficiency; random measurements of GH are not diagnostic if low and can only by useful to rule out a deficiency if high. Therefore, it is recommended that every patient with an abnormally low growth velocity should be tested with a GH stimulation test. Our data are insufficient to conclude which percentage of patients with 4H leukodystrophy would need a GH stimulation test; before we can make a general recommendation, further analysis of a larger cohort is required. As with any other child, height should be recorded and plotted at least once a year and more frequently in young children. GH treatment was reported to be ineffective in 2 patients who were treated in our cohort; however, it can be difficult to form conclusions based on these findings. GH treatment can often fail because of extraneous factors, such as nutrition, psychological aspects, and general state of health. Additionally, limitations of testing have been reported in establishing a firm diagnosis of GH deficiency, with a high rate of false-positive diagnoses reported in the literature (21). It is also possible that late sex steroid therapy could contribute to an impaired pubertal growth spurt and mislead the conclusion of an eventual lack of efficacy of GH treatment. Our findings support the early consultation and regular follow-up with an endocrinologist, especially in patients with short stature.

When analyzing growth data according to age group, it was found that young patients < 5 years of age were least affected by abnormal growth. This finding is expected given that the typical age of disease onset is during the second year of life. Growth was most affected in children 5 to 9 years of age, followed by children 10 to 14 years of age. We initially hypothesized that growth would be most affected in patients ≥ 15 years of age because of the frequent occurrence of delayed puberty. Per the recommendations for treatment, patients with delayed puberty are not usually treated before age 14; it is therefore possible that our cohort of patients age 5 to 9 years are most affected by growth abnormalities because older patients may have received sex steroids to stimulate puberty and growth, thereby compensating for the deficit caused by this disease. Indeed, in our cohort of patients ≥ 15 years of age, 22 patients received treatment of sex steroids and/or growth hormone. These treatments may have stimulated their growth and puberty. Therefore, the growth data of 22/57 patients may have been affected by a treatment that helped in compensating their short stature. It is clear that, as a whole, patients with 4H leukodystrophy are smaller compared to the general population within each analyzed age group. Thus, growth and height data should be collected by treating physicians so that abnormalities can be clearly identified, thereby facilitating more rapid treatment interventions. If growth anomalies are observed, GH stimulation tests should be considered. In future studies, it would be interesting to systematically follow growth and perform comparisons to bone age in individual patients to identify the subgroups of patients who would benefit most from GH replacement.

When analyzing head circumference data in our cohort of patients < 5 years of age, microcephaly seems to be more prevalent (9%) compared with the general population, in which there is a prevalence of 2% (22, 23). However, head circumference data were collected only for a limited number of patients. Microcephaly is typically not observed in the context of 4H leukodystrophy; only one case report describes this condition in 2 female siblings with a novel phenotype of 4H leukodystrophy with polymicrogyria and cataracts, who were also included in this study but not analyzed for head circumference because of a lack of head circumference measurements (24). As our results show an increased prevalence of small head size in the young age group < 5 years old, it would be interesting to further analyze additional data on head circumference to better characterize head size in 4H leukodystrophy.

A high percentage (41%) of patients with 4H leukodystrophy were found to have abnormal prolactin levels, where variability was seen in elevated (18%), or deficient (23%) levels. Limited information is available on the typical prolactin levels in patients with 4H leukodystrophy; 2 case reports have previously reported low prolactin levels in 2 patients, who were also included in this study (11, 12). Notably, prolactin values should be interpreted with the consideration of current treatments as some medications are known to cause hyperprolactinemia (e.g., psychoactive drugs). Because the full medication records of all patients in our cohort were not available, the interpretation of elevated results should be approached with caution. However, hypoprolactinemia does not commonly result from medications. It is known that the 2 previously published patients in our cohort with hypoprolactinemia (11, 12) were not under any treatments that could have interfered with testing, and their prolactin deficiency could not be explained by any known causes. Therefore, this finding provides foundation for the attribution of abnormal prolactin levels to 4H leukodystrophy itself.

Most patients in our cohort presented with normal random cortisol levels (20/21; 95%), except for one whose levels were elevated. ACTH levels were also normal for most patients tested (8/9, 89%). This suggests that adrenal dysfunction is not a common feature in our cohort; however, stimulation tests would be needed to fully conclude normal adrenal function in this patient population. Of note, random cortisol levels should be interpreted with caution, as cortisol secretion follows a circadian rhythm.

The prevalence of thyroid dysfunction appears to be increased in the 4H leukodystrophy population compared with the unaffected population. However, diverse abnormal variations of TSH and T4 hormones levels were observed in our cohort, suggesting that it is not associated with a typical disease phenotype. In the general pediatric population, hypothyroidism is the most common dysfunction with a prevalence of 0.135% in young people (< 22 years of age) (25). Compared with this, in our cohort, 4% of patients had hormone levels consistent with hypothyroidism.

Our results do not suggest a strong genotype-phenotype correlation between patients with biallelic pathogenic variants in the different genes associated with 4H leukodystrophy and short stature. However, a significant difference was observed between genotypes when considering delayed puberty. Patients with variants in *POLR3A* had the highest incidence of delayed puberty, followed by those with variants in *POLR3B*. Although no patients in our cohort with pathogenic variants in *POLR1C* demonstrated delayed puberty, data on only 4 patients were available and further analysis is required before definitive conclusions are formed. Additionally, because most patients in our cohort were compound heterozygous for different variants in each gene, and variants are located across different protein domains, it is difficult to make detailed phenotype-genotype correlations between specific pathogenic variants and clinical features.

It is important to be mindful that patients with 4H leukodystrophy are at risk for endocrine abnormalities, most commonly hypogonadotropic hypogonadism and GH deficiency. Thus, the treating physicians should have a high index of suspicion when signs or clinical symptoms appear. Measurements of relevant hormone levels and stimulation tests when necessary should be made to confirm the diagnosis. Finally, the decision of whether to treat should be evaluated on an individual basis while considering the advantages and disadvantages as no definitive recommendations currently exist.

In summary, endocrine abnormalities are underinvestigated in patients with 4H leukodystrophy. A future study is required to investigate the full extent and severity of typical endocrine abnormalities. Additional data on the evolution of growth, evaluated by regular height measurements, are necessary. Because hypodontia and teeth abnormalities are commonly seen in 4H leukodystrophy, it is also important to continue to monitor dental growth and the development of permanent tooth sets. An additional interesting aspect for future assessment could involve bone age and density and their correlations with growth development and puberty. To fully determine the prevalence of delayed puberty, clear information about the stage of puberty, age of menarche and breast development, level of testicular growth, and Tanner stage of development is needed. Additional data on endocrine hormone levels would also allow a more comprehensive evaluation of the prevalence of endocrine anomalies. In this study, data to evaluate the described growth and pubertal measures were only available for a limited number of patients. This presents a limitation and raises difficulty in drawing precise conclusions. In this sense, it is also possible that puberty and growth anomalies could have been recorded more commonly in patients with abnormal clinical findings, thus raising the frequency of these alterations in our cohort. With an expanded data set, a future objective would be to formulate evidence-based recommendations regarding the management of the endocrine manifestations of 4H leukodystrophy. In conclusion, this is the first study to systematically analyze endocrine abnormalities in a large cohort of patients affected by 4H leukodystrophy and provides the foundation for future comprehensive studies.

## Data Availability

The datasets generated during and/or analyzed during the current study are not publicly available but are available from the corresponding author on reasonable request.

## Abbreviations

CI: confidence interval
CNS: central nervous system
LHRH: luteinizing hormone releasing hormone
POLR3: RNA polymerase III

## Additional Information

***Disclosure Summary:*** A.V. receives grant and in-kind funding from Ionis, Illumina, Eli Lilly, Gilead, Biogen, Takeda, and Homology. B.P.v.d.W. receives research support from Radboud University Medical Centre, ZonMW, Hersenstichting, and Gossweiler Foundation. G.B. has received compensation for traveling to meetings and advisory boards from Ionis, Shire/Takeda, Children’s Hospital of Philadelphia, and Actelion Pharmaceuticals, served on the scientific advisory board for Ionis (2019), and has received research grants from Shire/Takeda and Bluebird Bio. G.M.H. received compensation for consulting with Kineticos. G.W. has received honoraria for speaking engagements from Desitin (Hamburg, Germany) and Novartis (Nuremberg, Germany), and gave scientific advice to PTC Therapeutics (Frankfurt, Germany). I.K.M. has received a travel grant from Shire/Takeda for an unrelated study. M.S.V.d.K. served on the scientific advisory board for Ionis (2019) and has received compensation for traveling to advisory boards from Ionis. M. Sylvain has received financial compensation for medical education conferences and/or has participated in advisory board for various pharmaceutical companies including Shire/Takeda, Janssen, Purdue, and Sunovion. N.I.W. has served on the scientific advisory board for Orchard and has received travel compensation. A. Blaschek, A. Bley, A. Hamati, A. Hill, A. Mirchi, A. Misbahuddin, A. Pizzino, A.D., A.M.I., A. Potic, A.R., A.S., B. Banwell, B. Brais, B.C., B.L.F., B.P., B.P.v.d.W., B.T.P.T., C. Tibussek, C.A.S., C.C., C.C.B., C.G., C.H., C.M.L., C.P., D. Timmann, D. Tonduti, D.B., D.G.B., D.G.M., D.P., D.R., E. Rampakakis, E. Rossignol, E.B., E.L., E.W., F.F., F.F.O., F.I.M., F.K.C., F.M., F.P., G.A.M.M., G.D., G.K., G.S., G.Y., H. Pedro, H. Philippi, H.M., H.O., I.C., I.H., I.T.M., J. Rankin, J. Rotteveel, J.F., J.G.A., J.L.B., J.R.O., K.B., K.G., L.K.S.R., L.S., L.T.T., M. Suri, M. Synofzik, M.D., M.E.G.G., M.F.R., M.K., M.M., M.N., M.P.M., M.V.L., N.R.E., N.S., N.U., P.G., P.K., P.S., P.S.P., P.W., R.F., R.I.W., R.L.P., R.M.L.v.S., R.P.J.M., R.S., R.S.C., S. Melançon, S. Moutton, S. Patzer, S. Pekic, S. Perrier, S. Saikali, S.M.K., S.N., S.O., S.T., S.V., T.A., T.d.G., U.K., V.P., W.B., W.M., and W.T.G. have nothing to declare.

## References

[1] Vanderver A , Prust M, Tonduti D, et al.; GLIA Consortium. Case definition and classification of leukodystrophies and leukoencephalopathies. Mol Genet Metab. 2015;114(4):494-500. 25649058 10.1016/j.ymgme.2015.01.006 PMC4390457

[2] van der Knaap MS , Bugiani M. Leukodystrophies: a proposed classification system based on pathological changes and pathogenetic mechanisms. Acta Neuropathol. 2017;134(3):351-382. 28638987 10.1007/s00401-017-1739-1 PMC5563342

[3] Schiffmann R , van der Knaap MS. Invited article: an MRI-based approach to the diagnosis of white matter disorders. Neurology. 2009;72(8):750-759. 19237705 10.1212/01.wnl.0000343049.00540.c8 PMC2677542

[4] Steenweg ME , Vanderver A, Blaser S, et al. Magnetic resonance imaging pattern recognition in hypomyelinating disorders. Brain. 2010;133(10):2971-2982. 20881161 10.1093/brain/awq257 PMC3589901

[5] Parikh S , Bernard G, Leventer RJ, et al.; GLIA Consortium. A clinical approach to the diagnosis of patients with leukodystrophies and genetic leukoencephelopathies. Mol Genet Metab. 2015;114(4):501-515. 25655951 10.1016/j.ymgme.2014.12.434 PMC4390485

[6] Bernard G , Vanderver A. POLR3-related leukodystrophy. In: Adam MP, Ardinger HH, Pagon RA, Wallace SE, Bean LJH, Mefford HC, Stephens K, Amemiya A, Ledbetter N, eds. GeneReviews. Seattle, WA: University of Washington Seattle; 2017. 22855961

[7] Wolf NI , Vanderver A, van Spaendonk RM, et al.; 4H Research Group. Clinical spectrum of 4H leukodystrophy caused by POLR3A and POLR3B mutations. Neurology. 2014;83(21):1898-1905. 25339210 10.1212/WNL.0000000000001002 PMC4248461

[8] La Piana R , Cayami FK, Tran LT, et al. Diffuse hypomyelination is not obligate for POLR3-related disorders. Neurology. 2016;86(17):1622-1626. 27029625 10.1212/WNL.0000000000002612 PMC4844237

[9] Vanderver A , Tonduti D, Bernard G, et al. More than hypomyelination in Pol-III disorder. J Neuropathol Exp Neurol. 2013;72(1):67-75. 23242285 10.1097/NEN.0b013e31827c99d2 PMC3797528

[10] Billington E , Bernard G, Gibson W, Corenblum B. Endocrine aspects of 4H leukodystrophy: a case report and review of the literature. Case Rep Endocrinol. 2015;2015:314594. 26113998 10.1155/2015/314594 PMC4465690

[11] Potic A , Brais B, Choquet K, Schiffmann R, Bernard G. 4H syndrome with late-onset growth hormone deficiency caused by POLR3A mutations. Arch Neurol. 2012;69(7):920-923. 22451160 10.1001/archneurol.2011.1963

[12] Potic A , Popovic V, Ostojic J, et al. Neurogenic bladder and neuroendocrine abnormalities in Pol III-related leukodystrophy. BMC Neurol. 2015;15:22. 25868523 10.1186/s12883-015-0283-7 PMC4351912

[13] La Piana R , Tonduti D, Gordish Dressman H, et al. Brain magnetic resonance imaging (MRI) pattern recognition in Pol III-related leukodystrophies. J Child Neurol. 2014;29(2):214-220. 24105487 10.1177/0883073813503902

[14] Vrij-van den Bos S , Hol JA, La Piana R, et al. 4H leukodystrophy: a brain magnetic resonance imaging scoring system. Neuropediatrics. 2017;48(3):152-160. 28561206 10.1055/s-0037-1599141

[15] Bernard G , Chouery E, Putorti ML, et al. Mutations of POLR3A encoding a catalytic subunit of RNA polymerase Pol III cause a recessive hypomyelinating leukodystrophy. Am J Hum Genet. 2011;89(3):415-423. 21855841 10.1016/j.ajhg.2011.07.014 PMC3169829

[16] Tétreault M , Choquet K, Orcesi S, et al. Recessive mutations in POLR3B, encoding the second largest subunit of Pol III, cause a rare hypomyelinating leukodystrophy. Am J Hum Genet. 2011;89(5):652-655. 22036172 10.1016/j.ajhg.2011.10.006 PMC3213403

[17] Thiffault I , Wolf NI, Forget D, et al. Recessive mutations in POLR1C cause a leukodystrophy by impairing biogenesis of RNA polymerase III. Nat Commun. 2015;6:7623. 26151409 10.1038/ncomms8623 PMC4506509

[18] Dorboz I , Dumay-Odelot H, Boussaid K, et al. Mutation in POLR3K causes hypomyelinating leukodystrophy and abnormal ribosomal RNA regulation. Neurol Genet. 2018;4(6):e289. 30584594 10.1212/NXG.0000000000000289 PMC6283457

[19] Delemarre-van de Waal HA . Application of gonadotropin releasing hormone in hypogonadotropic hypogonadism--diagnostic and therapeutic aspects. Eur J Endocrinol. 2004;151 Suppl 3:U89-U94. 15554892 10.1530/eje.0.151u089

[20] Orcesi S , Tonduti D, Uggetti C, Larizza D, Fazzi E, Balottin U. New case of 4H syndrome and a review of the literature. Pediatr Neurol. 2010;42(5):359-364. 20399393 10.1016/j.pediatrneurol.2010.01.015

[21] Grimberg A , DiVall SA, Polychronakos C, et al.; Drug and Therapeutics Committee and Ethics Committee of the Pediatric Endocrine Society. Guidelines for growth hormone and insulin-like growth factor-I treatment in children and adolescents: growth hormone deficiency, idiopathic short stature, and primary insulin-like growth factor-I deficiency. Horm Res Paediatr. 2016;86(6):361-397. 27884013 10.1159/000452150

[22] Abuelo D . Microcephaly syndromes. Semin Pediatr Neurol. 2007;14(3):118-127. 17980308 10.1016/j.spen.2007.07.003

[23] Sells CJ . Microcephaly in a normal school population. Pediatrics. 1977;59(2):262-265. 834509

[24] Jurkiewicz E , Dunin-Wąsowicz D, Gieruszczak-Białek D, et al. Recessive mutations in POLR3B encoding RNA polymerase III subunit causing diffuse hypomyelination in patients with 4H leukodystrophy with polymicrogyria and cataracts. Clin Neuroradiol. 2017;27(2):213-220. 26478204 10.1007/s00062-015-0472-1 PMC5487884

[25] Hunter I , Greene SA, MacDonald TM, Morris AD. Prevalence and aetiology of hypothyroidism in the young. Arch Dis Child. 2000;83(3):207-210. 10952634 10.1136/adc.83.3.207 PMC1718463

